# Comparative analysis of silage fermentation and *in vitro* digestibility of tropical grass prepared with *Acremonium* and *Tricoderma* species producing cellulases

**DOI:** 10.5713/ajas.18.0083

**Published:** 2018-05-31

**Authors:** Waroon Khota, Suradej Pholsen, David Higgs, Yimin Cai

**Affiliations:** 1 Faculty of Agriculture, Khon Kaen University, Khon Kaen 40002, Thailand; 2 Department of Biological and Environmental Sciences, University of Hertfordshire, AL10 9AB, UK; 3 Japan International Research Center for Agricultural Science (JIRCAS), Tsukuba, Ibaraki 305-8686, Japan

**Keywords:** Cellulase, *In vitro* Digestibility, Silage Fermentation, Tropical Grass

## Abstract

**Objective:**

To find out ways of improving fermentation quality of silage, the comparative analysis of fermentation characteristics and *in vitro* digestibility of tropical grasses silage applied with cellulases produced from *Acremonium* or *Tricoderma* species were studied in Thailand.

**Methods:**

Fresh and wilted Guinea grass and Napier grass silages were prepared with cellulases from *Acremonium* (AC) or *Trichoderma* (TC) at 0.0025%, 0.005%, and 0.01% on a fresh matter (FM), and their fermentation quality, chemical composition and *in vitro* digestibility were analyzed.

**Results:**

All silages of fresh Napier grass were good quality with lower pH, butyric acid, and ammonia nitrogen, but higher lactic acid content than wilted Napier grass and Guinea grass silage. Silages treated with AC 0.01% had the best result in terms of fermentation quality. They also had higher *in vitro* dry matter digestibility and *in vitro* organic matter digestibility at 6 and 48 h after incubation than other silages. Silages treated with lower levels at 0.005% or 0.0025% of AC and all levels of TC did not improve silage fermentation.

**Conclusion:**

The AC could improve silage fermentation and *in vitro* degradation of Guinea grass and Napier grass silages, and the suitable addition ration is 0.01% (73.5 U) of FM for tropical silage preparation.

## INTRODUCTION

Purple Guinea grass (*Panicum maximum* cv. TD 58) and Napier grass (*Pennisetum purpureum* ×*Pennisetum americanum* cv. Pak Chong 1) are widespread throughout tropical and subtropical areas. They adapt and grow well in a variety of soil types, are tolerant to different conditions and require quite low inputs for growth [1–3]. Both grasses are important roughage sources for ruminant feed because of high dry matter biomass production; ranged from 15 to 18 t/ha per year for Guinea grass [1] and 63 to 87 t/ha per year for Napier grass [3,4]. However, both grasses are seasonal with high dry matter yield over the short rainy season; thus, silage making is an increasingly important method to preserve fresh forages for feeding ruminants year-round [5]. It is well known to be difficult to make good quality silage with these species because they are usually low in water soluble carbohydrate (WSC) content, insufficient for rapid decrease of silage pH. They are also low in dry matter and high in fiber content which promotes the growth of clostridia and decreases nutrient utilization of ruminants [6–8].

Cellulase enzymes are popular biological products for improving the fermentation quality of silages. It is widely accepted that they can improve the fermentation process of both grasses and legumes and thus enhance cattle performance [6–8]. The two main reasons for cellulase enzyme addition in silage making are firstly, to enhance plant fiber degradation to increase the content of WSC as a lactic acid bacteria (LAB) substrate to produce lactic acid and enhance the fermentation process [9–11] and secondly, to break down the structural carbohydrate component thus improving silage digestibility [10,12,13]. Many microorganisms that produce various cellulolytic enzymes have been studied for several decades. The genus of *Acremonium* and *Trichoderma* has been especially famous for producing cellulolytic enzymes with relatively high enzymatic activity [14]. There is increasing interest in applying cellulase enzymes at ensiling due to reports that it can improve the fermentation quality of silage [15,16].

However, there is limited information available for comparative analysis of silage prepared with these cellulases produced from *Acremonium* and *Trichoderma*. The objectives of this study were to determine the fermentation quality and *in vitro* digestibility of tropical grass silage prepared with two cellulases at different concentration.

## MATERIALS AND METHODS

### Forage sample and silage preparation

Purple Guinea grass (*Panicum maximum* cv. TD 58) and Napier (*Pennisetum purpureum*×*Pennisetum americanum* cv. Pak Chong 1) grass were grown in a Korat soil series (Oxic Paleustults) at the experimental farm, Faculty of Agriculture, Khon Kaen University, Thailand in May 2014. Before planting, the plot for Guinea grass was ploughed twice and harrowed once. Root stock of purple Guinea grass was planted by hand with a spacing of 75×75 cm in row and between rows in a 10× 30 m of the rectangular plot. The plot was fertilized with cattle manure at a rate of 24,000 kg/ha which 4 separate equal portions of 6,000 kg/ha split applied for 4 cuts [16,17]. For Napier grass, one week before planting, the plot was ploughed and fertilized with basal fertilizers of NPK (15–15–15) and cattle manure at 300 and 12,500 kg/ha, respectively. The 11,111 stem cuttings of Napier grass were planted by hand with a spacing of 120×75 cm in row and between rows in a total area of 1,600 m^2^. Two weeks after planting, the plot was weeded out and nitrogen fertilizer (urea) applied at a rate of 60 kg/ha [4]. On 20 April 2015, guinea grass was cut to adjust the height to 10 cm above ground level and Napier grass was cut close to the soil surface. After 60 d of regrowth, both grasses were harvested in early morning and immediately chopped using a forage chopper (Supachai, Kanchanaburi, Thailand) to 1 cm particle size. Half of each chopped grass sample was wilted for 6 h in the shade to study the effect of moisture adjustment. Both fresh or wilted forages were then treated as follows: control (untreated), *Acremonium* cellulase (AC, Meiji Seika Pharma Co., Ltd, Tokyo, Japan): AC 0.0025%, AC 0.005%, AC 0.01%, *Trichoderma* cellulase (TC, Meiji Seika Pharma Co., Ltd, Japan): TC 0.0025%, TC 0.005%, and TC 0.01%. The production strain, main composition and carboxymethyl-cellulase (CMCase) of AC were *Acremonium cellulolyticus*, glucanase and pectinase, and 7,350 U/g; for TC they were *Tricoderma viride*, xylanase and glucanase, and 2,720 U/g, respectively. Treatments were ensiled using a small scale fermentation system [18]. Both AC and TC cellulases were added as % of fresh matter (FM). The design of this experiment was a 2 (two grasses)×2 (fresh vs wilted)×2 (two enzymes)×3 (three application levels) factorial arrangement in a completely randomized design with triplicates for each treatment. The silage was prepared with a laboratory-scale fermentation method [16]. The mixed grass with additive (1,000 g) was packed into a synthetic silo laminated from nylon and polyethylene (Hiryu KN type, Asahikasei, Tokyo, Japan), and sealed by using a vacuum sealer (SQ–303, Asahi Kasei Pax Corp., Japan). All silos were stored at room temperature (25°C to 37°C). On 30 d of ensiling, silos were opened for evaluation of fermentation products, chemical composition, and *in vitro* digestibility.

### Microorganism analysis

Microorganism numbers were analyzed using the plate count method as described by [19], and reported as colony-forming unit (cfu)/g of FM. Fresh or wilted forage samples (1,000 g) or silage samples (200 g) were sub-sampled for 10 g samples. Then, each sample (10 g) was homogenized with 90 mL sterilized distilled water, and serial dilutions in 0.85% sodium chloride solution at 10^−1^ to 10^−5^. Each dilution (20 μL) was dropped and spread on prepared agar plates. MRS agar (Difco Laboratories, Detroit, MI, USA) was used for the LAB count, which the agar plates were put into an anaerobic box (TE–HER Hard Anaerobox, ANX–1; Hirosawa Ltd., Tokyo, Japan), and incubation at 30°C for 48 h in an incubator. Blue light broth agar (Nissui-seiyaku Ltd., Tokyo, Japan) was used for coliform bacteria counts after incubating at 30°C for 48 h. Nutrient agar (Difco, USA) and potato dextrose agar (Nissui-seiyaku, Japan) were used for aerobic bacteria, yeast, and mold. Mold was counted after 2 d of incubation. Yeasts were distinguished from molds or bacteria by colony appearance and cell morphology observation.

### Chemical composition

Cold water extraction of silage samples was used for fermentation end-product analysis following the method of Cai [20]. Ten grams of wet silage was soaked with 90 mL sterilized distilled water, and then, incubated at 4°C in a refrigerator overnight. Silage pH was measured immediately after incubating using a glass electrode pH meter (FiveGo; Mettler Toledo, Greifensee, Switzerland), and 10 mL of cold water extract was sampled and stored at −20°C for further analysis of organic acids and ammonia nitrogen concentrations. Organic acid content of silages was measured by HPLC methods [20]. Ammonia nitrogen concentration was analyzed using a spectrophotometer (UV/VIS Spectrometer, PG Instruments Ltd., London, UK) [21]. The lactate buffering capacity (LBC) of forages was analyzed by the titration method viz. 10 g of sample homogenized with distilled water, titrated with 0.1 M hydrochloric acid (HCl) to decrease pH from initial pH to 3, then titrated to pH 6 with 0.1 M sodium hydroxide (NaOH) [13]. The WSC content of forage was extracted and measured following the methods of [16].

Forage and silage samples on 30 d of ensiling were dried at 60°C for 48 h in a hot air oven, and ground through a 1 mm mesh screen. Dry matter (DM), organic matter (OM), crude protein (CP), and ether extract (EE) were analyzed following methods 934.01, 942.05, 976.05, and 920.39, respectively [22]. Neutral detergent fiber (NDF), acid detergent fiber (ADF), and acid detergent lignin (ADL) were analyzed using a fiber analyzer (ANKOM 200, ANKOM Technology, New York, USA), based on the method of [23].

### Animal care

The animal experiment was approved by the Animal Care and Use Committee of Khon Kaen University (KKU), Khon Kaen, Thailand and performed at KKU in August 2015. The experiment was performed according to recommendations proposed by the European Commission [24] and to minimize the suffering of animals.

### *In vitro* digestibility analysis

*In vitro* digestibility of DM (IVDMD) and OM (IVOMD) were measured after incubating samples in buffered rumen fluid at 6 and 48 h. The buffered rumen inoculum was prepared following the method of [25]. Rumen fluid was collected from two dairy steers by a stomach tube sucker before morning feeding. The pH of rumen fluid was 7.2. Ground silage samples (0.5 g) was put into serum bottles of 50 mL capacity (3 replications per sample). The bottles were closed by a rubber stopper with an aluminum seal cap. The buffered rumen inoculum (40 mL) was injected into each sample bottle using a 60 mL syringe (Nipro Thailand corporation, Ltd., Phra Nakhon Si Ayutthaya, Thailand) with 18 gauge×3.5 cm needle (Nipro Corporation, Osaka, Japan), and flushed with carbon dioxide gas to produce an anaerobic condition. All samples were incubated in a water bath at 39°C, swirled by hand and the head space gas production released using a 21 gauge×3.5 cm needle (Nipro, Japan) at 2 h intervals. Two blanks of 40 mL of rumen inoculum per bottle were also incubated.

The animals were housed in individual pen equipped with mineral blocks (FNZ Red Lick, Thai Serve Co. Ltd., Buriram, Thailand; mineral composition: NaCl 930.00 g, Mg 2.00 g, Zn 0.77 g, Mn 0.50 g, Co 18.00 g, I 0.05 g, Se 0.01 g, Cu 0.22 g, another 2.50 g) and fresh water, and fed concentrate (16% CP, 11.0 MJ/kg of metabolizable energy, feed ingredient including: 10% palm kernel meal, 7% coconut meal, 6% rice bran, 70% cassava chip, 4% urea, 1% sulfur, 1% mineral premix, and 1% salt on a DM basis) at 0.5% of body weight and *ad libitum* rice straw daily. The diet was fed in two equal meals at 08:00 and 16:00 h.

### Statistical analysis

Data on fermentation products, chemical composition, and *in vitro* digestibility of silages at 30 d after fermentation were analyzed using a completely randomized design with a 2×2× 2×3 (forages [A]×moisture adjustment [B]×enzymes [C]× application levels [D]) factorial treatment combinations. The analysis of variance procedure of SAS version 6.12 (SAS Institute Inc., Cary, NC, USA) was used for the analysis and the statistical model is as follows:

$${\text{Y}}_{\text{ijklm}}=\mu +\alpha_{\text{i}}+\beta_{\text{j}}+\gamma_{\text{k}}+\delta_{1}+\alpha \beta \gamma \delta_{\text{ijkl}}+{ɛ}_{\text{ijklm}}$$

Where Y_ijklm_ = observation; μ = overall mean, α_i_ = forage effect (i = Guinea grass and Napier grass), β_j_ = moisture adjustment effect (j = fresh and wilted), γ_k_ = enzyme effect (AC and TC), δ_l_ = application levels (0.0025%, 0.005%, and 0.01%), αβγδ_ijkl_ = forages×moisture adjustment×enzymes×application levels, and ɛ_ijklm_ = error. The significant difference among treatment means was tested by Duncan’s new multiple range test at p = 0.05 [26].

## RESULTS

### Microorganism population and chemical composition of forages

The microorganism counts of forages are shown in Table 1. Fresh Guinea grass and fresh Napier grass showed similar numbers of LAB (10^3^ cfu/g of FM), aerobic bacteria (10^7^ cfu/g of FM), and coliform bacteria (10^7^ cfu/g of FM). The counts of yeasts and molds of both fresh grasses ranged from 10^4^ to 10^5^ and 10^2^ to 10^3^ cfu/g of FM, respectively. After wilting process, the counts of LAB, yeasts and molds of both grasses increased from 10^4^ to 10^5^, 10^5^ to 10^6^, and 10^4^ cfu/g of FM, respectively, the counts of coliform bacteria and aerobic bacteria did not change by the wilting process.

The chemical composition and LBC of Guinea grass and Napier grass are shown in Table 2. The OM content was higher (p<0.01) and WSC content was lower (p<0.01) in Napier grasses than those in Guinea grasses. During the wilting process, the DM of Guinea grass and Napier grass significantly (p<0.01) increased by 7% and 8%, respectively, but the contents of ADL and LBC contents did not differ. Compared with fresh Guinea grass, the contents of CP and ADF were higher (p<0.01), but the NDF content was lower in wilting Guinea grass. Also, wilted Napier grass showed higher (p<0.01) CP content and lower EE content than fresh Napier grass.

### Fermentation quality of silages

The DM, pH, and fermentation products of Guinea grass and Napier grass silages at 30 d of ensiling are shown in Table 3. The interaction between A, B, C, and D influenced (p<0.01) the silage DM, pH, all organic acid contents, and ammonia nitrogen content. Guinea grass silage treated with AC 0.01% showed good quality with lower (p<0.01) pH and ammonia nitrogen, and higher (p<0.01) lactic acid content than other silages. Regarding the differences in production strain (*A. cellulolyticus* vs *T. viride*), main composition (glucanase and pectinase, vs xylanase and glucanase), and CMCase (7,350 vs 2,720 U/g) between AC and TC, the highest (p<0.01) pH and ammonia nitrogen content with lowest (p<0.01) lactic acid content were found in wilted Guinea grass silages treated with TC 0.05%. The AC treatments improved grass silage quality more than TC treatments. The addition level of AC at 0.01% improved fermentation quality more than 0.005% and 0.0025%.

### Microorganism counts of silages

The microbiological analysis of Guinea grass and Napier grass silages at 30 d of ensiling are shown in Table 4. The A, B, C, D and their interaction (A×B×C×D) were not significantly different (p<0.05) in LAB count, aerobic bacteria and yeasts. Coliform bacteria and molds were below the detectable level (10^2^ cfu/g of FM) in all silages. The LAB population of all silages was dominant ranging from 10^7^ to 10^8^ cfu/g of FM. Aerobic bacteria were 10^3^ to 10^4^ cfu/g of FM and yeast were 10^2^ to10^4^ cfu/g of FM.

### Chemical composition of silages

The chemical compositions of Guinea grass and Napier grass silages at 30 d of ensiling are shown in Table 5. The significances of A×B×C×D (p<0.001) influenced all chemical compositions. The OM content of AC or TC treated Guinea grass silage were higher (p<0.01) than other silages. The wilted Napier grass silages treated with AC 0.01% showed higher (p<0.01) CP content and lower (p<0.01) NDF and ADF content than other silages. The highest (p<0.01) fiber contents were observed in TC treated fresh Guinea grass silages. When silages treated with AC, the CP, EE, and ADL contents were significantly (p< 0.01) higher and the NDF and ADF contents were significantly (p<0.01) lower than TC treatments; and the application levels of enzymes at 0.01% decreased fiber content more than 0.005% and 0.0025%.

### *In vitro* digestibility of silages

The *in vitro* digestibility of Guinea grass and Napier grass silages at 30 d of ensiling are shown in Table 6. Fresh Napier grass silages treated with AC 0.01% had higher (p<0.05) IVDMD than other treatments. Napier grass silages had higher (p<0.01) IVDMD and IVOMD than Guinea grass silages. The wilted grasses silages had higher (p<0.05) IVOMD than fresh grasses silages. When silages treated with AC, the IVDMD and IVOMD at 6 h after incubation were significantly higher (p<0.01) than those of TC treatments. The IVDMD and IVOMD of 0.01% cellulase treated silage at 6 and 48 h after incubation were greater (p<0.05) than those of 0.0025%. The significances of A×B×C×D (p<0.05) influenced IVDMD at 48 h after incubation in buffered rumen fluid, while IVDMD at 6 h after incubation and IVOMD at both 6 and 48 h after incubation did not.

## DISCUSSION

Generally, the DM content of forages influences fermentation quality of the silage; and optimal DM content ranges from 30% to 40% for good quality silage making [13,27]. If dry matter content is less than 20%, the fermentation process would be dominated by clostridium, resulting in low quality silage production [28]. In this study, the DM content of fresh Guinea grass and Napier grass were lower than 18%, which was not ideal to preserve the forage. When both grasses were wilted for 6 h in the field, DM contents of Guinea grass and Napier grass were higher than 21%, much more suitable for ensiling. The decreasing of moisture during the wilting process under this experiment is negligible and less than those of the optimal DM content ranges. In addition, the wilted both grasses as shown Table 2 had a higher CP and lower ADF compared with that of fresh forage. However, we cannot fully explain the mechanism of these effects, this may be influenced by the climatic conditions such as low temperature and high humidity during the wilting process. Future study is necessary to study the effect of climatic condition on the chemical composition of forage during wilting process.

The CP, NDF, and ADF of Guinea grass and Napier grass used in this study were similar to our previous work which reported that Guinea grass and Napier grass harvested at 60 d of regrowth were lower in CP contents, but high in fiber [16]. The WSC in the ensiling material also plays an important role in silage fermentation and it is the main energy source for LAB growth to produce lactic acid [29,30]. Wilkinson [27] suggested that WSC needs to be greater than 10% of DM for good fermentation. However, the WSC contents of fresh forages in this present study were less than 2.7% of DM (Table 2). Although a lower WSC content was found in Guinea grass compared to Napier grass, it would be difficult to make good quality silage from both grasses [5].

At 30 d of fermentation, Guinea grass silages were poor quality. As shown in Tables 1, 2, both fresh and wilted Guinea grasses had low LAB counts and WSC content which were not sufficient to produce more lactic acid for good fermentation. Thus, the pH of Guinea grass silages did not decrease below 4.2, which cannot inhibit the growth of harmful bacteria, especially clostridia [13]. As a result, a harmful fermentation could occur with clostridia using WSC, amino acid, and other organic acids to produce butyric acid and ammonia nitrogen.

Some protein of forages breaks down to simpler or non-protein nitrogen during wilting process, result in decreased true protein solubility [31]. Nevertheless, this is consistent with previous studies [32] that total protein content of intact asparagus spear and excised tip sections increased approximately 10% after 6 to 12 h of harvested. Also, our results indicated that wilted forages silages were lower (p<0.01) lactic acid content than fresh forage silages. Consistently, our previous study [16] evidenced a lower lactic acid concentration in silages prepared from wilted forages compared to fresh forages. The significantly higher DM in wilted forages silages may attribute to decrease LAB growth and activity during silage fermentation.

When grass silages treated with AC 0.01%, their fermentation quality were more improved compared to other treatments. Our findings are in agreement with [9,33–35]. that the addition of cellulase enzyme resulted in a decrease of pH and increased lactic acid content in sorghum straw, oil palm frond and mixed hulless-barley straw with corn silages. This could be attributed to the cellulase enzyme degrading plant cellulose so increasing the WSC, an essential substrate for LAB growth and more lactic acid production. As a result, the pH of silages decrease rapidly inhibiting the growth of clostridia and preventing proteolysis in the silo [33,36]. Some studies [15,29,34,37] reported that addition of cellulase resulted in a decrease in fiber content of mixed silages of hulless-barley straw and corn, wheat straw, *Leymus chinensis*, and Napier grass silage. On the other hand, cellulase addition had no effect on NDF and ADF content of barley and vetch silage [38]. In the present study, the AC improved silage fermentation more than TC, and the AC 0.01% cellulase effectively increased CP content and reduced NDF and ADF contents of silages. Perhaps the type, composition and enzyme activity of cellulase may affect cellulose degradation and silage fermentation. Generally, cellulases catalyze the hydrolysis of cellulose, which are mainly three types: endoglucanases, cellobiohydrolases and β-glucosidases, and it is used for any occurring mixture or complex of various such enzymes, that act serially or synergistically to decompose cellulosic material. *Acremonium cellulolyticus*-producing cellulases contain a strong glucanase and pectinase, and cellulase produced by *Trichoderma viride* contains mainly xylanase and glucanase. Therefore, *Acremonium cellulolyticus*-producing cellulase is said to be more potent than *Trichoderma viride*-producing cellulase. This indicated that AC is more effective for improvement of silage fermentation than TC, and its addition concentration is necessary to be 0.01% on a FM basis.

The digestibility of roughage also plays an important role in animal production [10,29]. The IVDMD and IVOMD depend on physical characteristics of forage, especially the fiber content, low NDF and ADF contents resulted in a rapid increase in digestibility of DM and OM [29,39]. In the present study, Napier grass silages had higher IVDMD and IVOMD when compared to Guinea grass. This could be attributed to the differences in chemical composition of Guinea grass and Napier grass. Guinea grass had higher fiber content than Napier grass as shown in Table 2. Furthermore, the cellulase enzyme addition, especially AC 0.01% treated silages had greater IVDMD and IVOMD values than other enzyme application levels in both 6 and 48 h of incubation (Table 6). This study is consistent with previous studies reported by [10,40] that the addition of an exogenous cellulase enzyme could increase IVDMD of forage crops, but inconsistent with [29] that cellulase addition did not increase the IVDMD of *Leymus chinensis* after 48 h of incubation. The greater effect of AC 0.01% treatment in the present study due to the composition of enzymes. The AC used in this study has both glucanase and pectinase; the synergistic effects of these enzymes probably increase silage digestibility. The CMCase activity is also higher in AC (7,350 U/g) than TC (2,720U/g).

These results confirmed that Napier grass has more suitable ensiling characteristics than Guinea grass, and the wilted silage can decrease the feed nutrient loss and improve the *in vitro* digestibility. The addition of AC 0.01% cellulase (73.5 U/g of FM) was effective in improving the silage fermentation and promoting the *in vitro* degradation of tropical grass.

## CONCLUSION

Silage fermentation and *in vitro* digestibility of Guinea grass and Napier grass prepared with cellulase enzyme at different addition concentration were studied. The AC has the potential to improve the fermentation quality, chemical composition and *in vitro* degradation of Guinea grass and Napier grass, but the TC did not affect the tropical silage fermentation. The AC addition at 0.01% (73.5 U/g) of FM was the most promising for improving silage fermentation containing low WSC.

## Figures and Tables

**Table 1:** Microorganism counts (cfu/g of FM) of Guinea grass and Napier grass before ensiling

Items	Lactic acid bacteria	Coliform bacteria	Aerobic bacteria	Yeast	Mold
Guinea grass					
Fresh	1.8×10^3^	1.5×10^7^	2.6×10^7^	2.9×10^4^	1.3×10^3^
Wilted	1.2×10^4^	4.2×10^7^	2.4×10^7^	1.5×10^5^	1.6×10^4^
Napier grass					
Fresh	2.5×10^3^	8.1×10^7^	1.1×10^7^	1.5×10^5^	2.5×10^2^
Wilted	3.5×10^5^	6.2×10^7^	2.0×10^7^	2.0×10^6^	2.9×10^4^

cfu, colony-forming unit; FM, fresh matter.

**Table 2:** Chemical composition (% of DM) and lactate buffer capacity (LBC, mE/kg of DM) of Guinea grass and Napier grass before ensiling

Items	DM	OM	CP	EE	NDF	ADF	ADL	WSC	LBC
Guinea grass									
Fresh	17.81^c^	90.16^a^	7.49^c^	1.65^b^	77.47^a^	48.26^b^	3.32	0.48^b^	760.81
Wilted	24.78^a^	89.76^a^	8.21^b^	1.63^b^	76.24^b^	51.45^a^	2.76	0.39^b^	526.64
Napier grass									
Fresh	13.08^d^	87.23^b^	7.19^c^	2.07^a^	71.14^c^	42.81^c^	3.18	2.59^a^	722.48
Wilted	21.86^b^	87.62^b^	8.70^a^	1.66^b^	70.82^c^	39.11^d^	2.92	2.46^a^	622.15
SEM	1.020	0.154	0.119	0.091	0.237	0.452	3.32	0.058	75.167
p-value	0.001	<0.001	<0.001	0.022	<0.001	<0.001	0.411	<0.001	0.110

DM, dry matter; OM, organic matter; CP, crude protein; EE, ether extract; NDF, neutral detergent fiber; ADF, acid detergent fiber; ADL, acid detergent lignin; WSC, water soluble carbohydrate; SEM, standard error of the mean.

a–d Means within columns with different superscripts differ at p<0.05.

**Table 3:** Dry matter (%), pH and fermentation products (g/kg of DM) of Guinea grass and Napier grass silages at 30 d after fermentation

Treatments	DM	pH	Lactic acid	Acetic acid	Propionic acid	Butyric acid	Ammonia nitrogen
Fresh Guinea							
AC 0.0025%	18.43^c^	4.56^bc^	0.10^h^	4.42^b^	0.21^b^	3.09^a^	0.99efgh
AC 0.005%	17.09^cd^	4.49^c^	0.52^gh^	3.92^c^	0.13^c^	3.28^a^	0.88fghi
AC 0.01%	18.34^c^	3.55^i^	6.71^ab^	1.79^ef^	0.02efg	0.18defg	0.18^j^
TC 0.0025%	17.09^cd^	4.59^bc^	0.00^h^	5.27^a^	0.32^a^	2.66^b^	1.49bcde
TC 0.005%	18.18^c^	4.63^bc^	0.01^h^	4.71^b^	0.35^a^	2.23^c^	1.20cdef
TC 0.01%	17.60^c^	4.60^bc^	0.00^h^	5.18^a^	0.21^b^	2.62^b^	1.63bcd
Wilted Guinea							
AC 0.0025%	23.84^a^	4.66^bc^	1.33efgh	1.15^hi^	0.03efg	0.18defg	1.78^bc^
AC 0.005%	23.46^a^	4.49^c^	2.78def	1.74^fg^	0.03efg	0.42^d^	1.56bcde
AC 0.01%	23.48^a^	4.25^de^	2.07efgh	0.88^i^	0.02efg	0.06efg	1.69^bc^
TC 0.0025%	24.07^a^	4.90^a^	1.75efgh	2.31^d^	0.06^d^	0.24defg	2.08^b^
TC 0.005%	23.29^a^	5.05^a^	0.47^gh^	1.29fghi	0.05^de^	0.42^d^	2.74^a^
TC 0.01%	23.65^a^	4.91^a^	0.71fgh	1.18^hi^	0.04def	0.21defg	1.74^bc^
Fresh Napier							
AC 0.0025%	13.51^ef^	3.89^gh^	2.69defg	1.55fgh	0.02efg	0.01^g^	0.42ghij
AC 0.005%	12.64^f^	3.78^h^	7.56^a^	1.35fghi	0.00^g^	0.03^fg^	0.32^ij^
AC 0.01%	15.06^de^	3.87^gh^	5.52^ab^	1.21ghi	0.01^fg^	0.03^fg^	0.27^j^
TC 0.0025%	18.08^c^	4.23^de^	4.73bcd	1.68fgh	0.01^fg^	0.04^fg^	0.76fghij
TC 0.005%	14.18^ef^	4.08^ef^	5.51^ab^	1.47fgh	0.01^fg^	0.04^fg^	0.65fghij
TC 0.01%	13.79^ef^	4.03^fg^	5.36^bc^	1.22ghi	0.00^g^	0.02^g^	0.40hij
Wilted Napier							
AC 0.0025%	23.46^a^	4.33^d^	0.76fgh	1.57fgh	0.01^fg^	0.01^g^	0.74fghij
AC 0.005%	22.52^ab^	4.17def	4.85bcd	1.48fgh	0.01^fg^	0.01^g^	0.67fghij
AC 0.01%	23.58^a^	4.09^ef^	5.07^bc^	1.42fgh	0.00^g^	0.01^g^	0.77fghij
TC 0.0025%	22.08^ab^	4.68^b^	3.27cde	2.48^d^	0.02efg	0.35^d^	1.03efg
TC 0.005%	23.61^a^	4.64^bc^	3.26cde	2.23^de^	0.01^fg^	0.08efg	1.04^ef^
TC 0.01%	21.06^b^	4.68^b^	3.19cde	2.51^d^	0.00^g^	0.33def	1.07def
SEM	0.826	0.062	0.781	0.188	0.012	0.105	0.210
Grasses means							
Guinea grass	20.71^a^	4.56^a^	1.37^b^	2.82^a^	0.12^a^	1.30^a^	1.50^a^
Napier grass	18.63^b^	4.21^b^	4.31^a^	1.68^b^	0.01^b^	0.08^b^	0.68^b^
Moisture adjust							
Fresh	16.17^b^	4.19^b^	3.23^a^	2.82^a^	0.11^a^	1.19^a^	0.77^b^
Wilted	23.18^a^	4.57^a^	2.46^b^	1.69^b^	0.02^b^	0.19^b^	1.41^a^
Enzymes							
AC	19.62	4.18^b^	3.33^a^	1.87^b^	0.04^b^	0.61^b^	0.86^b^
TC	19.73	4.58^a^	2.35^b^	2.63^a^	0.09^a^	0.77^a^	1.32^a^
Application levels							
0.0025%	20.07	4.48^a^	1.83^c^	2.56^a^	0.09^a^	0.82^a^	1.16
0.005%	19.37	4.42^b^	3.12^b^	2.27^b^	0.07^b^	0.81^a^	1.13
0.01%	19.57	4.25^c^	3.58^a^	1.92^c^	0.04^c^	0.43^b^	0.97
Significance of main effect and interaction							
Grasses means (A)	<0.001	<0.001	<0.001	<0.001	<0.001	<0.001	<0.001
Moisture adjust (B)	<0.001	<0.002	0.008	0.008	<0.001	<0.001	<0.001
Enzymes (C)	0.712	<0.003	0.001	0.001	<0.001	<0.001	<0.001
Application levels (D)	0.141	<0.004	<0.001	<0.001	<0.001	<0.001	0.087
A×B×C×D	0.001	<0.005	<0.001	<0.001	<0.001	<0.001	0.007

DM, dry matter; AC, *Acremonium* cellulase (Meiji Seika Pharma Co. Ltd., Tokyo, Japan); TC, *Trichoderma* cellulase (Meiji Seika Pharma Co. Ltd., Japan); SEM, standard error of the mean.

a–j Means within columns with different superscripts differ at p<0.05.

Values are means of three silage samples.

**Table 4:** Microbiological analysis of Guinea grass and Napier grass silages at 30 d after fermentation

Treatments	Microorganism (cfu/g of FM)				

	Lactic acid bacteria	Coliform bacteria	Aerobic bacteria	Yeast	Mold
Fresh Guinea					
AC 0.0025%	9.0×10^7^	ND	1.4×10^4^	ND	ND
AC 0.005%	3.9×10^8^	ND	1.2×10^4^	ND	ND
AC 0.01%	2.8×10^8^	ND	1.5×10^4^	ND	ND
TC 0.0025%	1.2×10^8^	ND	3.5×10^3^	1.9×10^2^	ND
TC 0.005%	7.9×10^7^	ND	0.9×10^4^	1.7×10^2^	ND
TC 0.01%	2.6×10^8^	ND	1.9×10^4^	2.3×10^2^	ND
Wilted Guinea					
AC 0.0025%	2.4×10^8^	ND	8.1×10^3^	ND	ND
AC 0.005%	7×10^7^	ND	1.6×10^4^	ND	ND
AC 0.01%	1.5×10^8^	ND	8.0×10^3^	ND	ND
TC 0.0025%	1.2×10^8^	ND	6.8×10^3^	4.6×10^2^	ND
TC 0.005%	3.7×10^8^	ND	1.8×10^4^	5.6×10^2^	ND
TC 0.01%	1.7×10^8^	ND	1.6×10^4^	1.9×10^2^	ND
Fresh Napier					
AC 0.0025%	3.0×10^7^	ND	1.8×10^4^	ND	ND
AC 0.005%	1.6×10^8^	ND	3.4×10^4^	7.2×10^2^	ND
AC 0.01%	1.5×10^8^	ND	1.6×10^4^	1.0×10^2^	ND
TC 0.0025%	4.7×10^7^	ND	8.0×10^4^	ND	ND
TC 0.005%	1.5×10^8^	ND	1.0×10^4^	1.4×10^2^	ND
TC 0.01%	1.6×10^8^	ND	7.8×10^3^	1.6×10^2^	ND
Wilted Napier					
AC 0.0025%	8.1×10^7^	ND	7.8×10^3^	1.0×10^4^	ND
AC 0.005%	1.8×10^8^	ND	2.1×10^4^	1.0×10^3^	ND
AC 0.01%	1.4×10^8^	ND	1.5×10^4^	4.9×10^3^	ND
TC 0.0025%	7.6×10^8^	ND	6.6×10^3^	7.8×10^3^	ND
TC 0.005%	1.0×10^8^	ND	4.7×10^3^	5.1×10^3^	ND
TC 0.01%	7.3×10^8^	ND	2.6×10^4^	1.4×10^4^	ND
SEM	1.331	ND	2.037	57.895	ND
Grasses means					
Guinea grass	1.9×10^8^	ND	1.2×10^4^	1.6×10^2^	ND
Napier grass	1.1×10^8^	ND	2.1×10^4^	4.8×10^3^	ND
Moisture adjust					
Fresh	1.6×10^8^	ND	1.9×10^4^	1.3×10^2^	ND
Wilted	1.4×10^8^	ND	1.3×10^4^	3.7×10^3^	ND
Enzymes					
AC	1.6×10^8^	ND	1.5×10^4^	1.4×10^3^	ND
TC	1.4×10^8^	ND	1.7×10^4^	3.6×10^3^	ND
Application levels					
0.0025%	1.0×10^8^	ND	1.8×10^4^	2.3×10^3^	ND
0.005%	1.9×10^8^	ND	1.6×10^4^	2.7×10^3^	ND
0.01%	1.7×10^8^	ND	1.5×10^4^	2.5×10^3^	ND
Significance of main effect and interaction					
Grasses means (A)	0.088	ND	0.239	0.250	ND
Moisture adjust (B)	0.779	ND	0.341	0.243	ND
Enzymes (C)	0.675	ND	0.790	0.294	ND
Application levels (D)	0.271	ND	0.943	0.991	ND
A×B×C×D	0.939	ND	0.660	0.878	ND

FM, fresh matter; ND, not detected; AC, *Acremonium cellulase* (Meiji Seika Pharma Co. Ltd., Tokyo, Japan); TC, *Trichoderma cellulase* (Meiji Seika Pharma Co. Ltd., Japan); SEM, standard error of the mean.

Values are means of 3 silage samples.

**Table 5:** Chemical compositions (% of DM) of Guinea grass and Napier grass silages at 30 d after fermentation

Treatments	OM	CP	EE	NDF	ADF	ADL
Fresh Guinea						
AC 0.0025%	90.91^a^	6.64^ef^	2.85abcde	75.58^ab^	50.29cde	4.18bcdef
AC 0.005%	90.54^a^	6.26^fg^	2.78abcde	74.82^b^	49.54^de^	4.57abcd
AC 0.01%	90.83^a^	6.76^de^	2.36^fg^	71.04^de^	46.17^f^	3.91cdefgh
TC 0.0025%	90.51^a^	5.84hij	2.72abcdefg	75.36^ab^	51.84^ab^	5.22^a^
TC 0.005%	90.31^a^	6.26^fg^	2.64cdefg	75.12^ab^	50.97^bc^	4.34abcde
TC 0.01%	90.60^a^	5.69ijk	2.68bcdefg	76.48^a^	52.67^a^	4.87^ab^
Wilted Guinea						
AC 0.0025%	89.18^b^	7.33^bc^	2.92abcd	70.69^e^	49.63^de^	2.64^j^
AC 0.005%	89.16^b^	7.34^bc^	2.97abc	70.30^e^	49.12^e^	4.14bcdef
AC 0.01%	89.26^b^	6.91^de^	3.08^ab^	68.46^f^	47.31^f^	4.67abc
TC 0.0025%	89.05^b^	7.05^cd^	2.68bcdefg	72.22^cd^	50.56bcd	3.99bcdefg
TC 0.005%	89.01^b^	7.64^b^	2.84abcde	72.52^c^	50.67bcd	3.54efghij
TC 0.01%	89.28^b^	7.71^b^	3.09^ab^	72.42^cd^	51.42abc	2.92^ij^
Fresh Napier						
AC 0.0025%	86.05^d^	5.85hij	2.74abcdef	65.27^ij^	41.62^h^	3.69defghi
AC 0.005%	87.22^c^	5.56^jk^	2.98abc	67.64^fg^	43.01^g^	4.31bcde
AC 0.01%	86.77^c^	5.97ghi	3.02abc	66.57ghi	42.10^gh^	4.68abc
TC 0.0025%	85.72^d^	6.14^gh^	2.75abcdef	67.10fgh	43.03^g^	2.83^ij^
TC 0.005%	84.59^e^	5.55^jk^	2.32^g^	65.24^ij^	42.01^gh^	2.65^j^
TC 0.01%	85.84^d^	5.34^k^	2.46efg	66.29ghi	43.17^g^	2.81^ij^
Wilted Napier						
AC 0.0025%	85.46^d^	7.67^b^	2.90abcd	64.49^jk^	38.82^j^	3.02hij
AC 0.005%	85.39^d^	7.71^b^	2.69bcdefg	63.34^kl^	37.97^jk^	3.37fghij
AC 0.01%	85.89^d^	8.25^a^	2.89abcd	62.79^l^	36.89^k^	3.28fghij
TC 0.0025%	85.98^d^	7.59^b^	2.71abcdefg	64.32^jk^	40.05^i^	2.85^ij^
TC 0.005%	85.50^d^	7.51^b^	3.12^a^	66.42ghi	40.26^i^	3.04hij
TC 0.01%	85.50^d^	7.64^b^	2.53defg	66.09^hi^	40.28^i^	3.17ghij
SEM	0.265	0.148	0.141	0.551	0.489	0.322
Grasses means						
Guinea grass	89.89^a^	6.79	2.80	72.92^a^	50.02^a^	4.08^a^
Napier grass	85.83^b^	6.73	2.76	65.46^b^	40.77^b^	3.31^b^
Moisture adjust						
Fresh	88.32^a^	5.99^b^	2.69^b^	70.54^a^	46.37^a^	4.00^a^
Wilted	87.39^b^	7.53^a^	2.87^a^	67.84^b^	44.41^b^	3.39^b^
Enzymes						
AC	88.06^a^	6.85^a^	2.85^a^	68.42^b^	44.37^b^	3.87^a^
TC	87.66^b^	6.66^b^	2.71^b^	69.97^a^	46.41^a^	3.52^b^
Application levels						
0.0025%	87.86^a^	6.77	2.78	69.38^a^	45.73^a^	3.55
0.005%	87.72^b^	6.73	2.79	69.43^a^	45.44^a^	3.74
0.01%	87.99^a^	6.78	2.76	68.77^b^	45.00^b^	3.79
Significance of main effect and interaction						
Grasses means (A)	<0.001	0.306	0.396	<0.001	<0.001	<0.001
Moisture adjust (B)	<0.001	<.001	0.001	<0.001	<0.001	<0.001
Enzymes (C)	<0.001	0.001	0.009	<0.001	<0.001	0.003
Application levels (D)	0.059	0.666	0.881	0.013	0.005	0.207
A×B×C×D	<0.001	<0.001	0.002	<0.001	<0.001	<0.001

DM, dry matter; OM, organic matter; CP, crude protein; EE, ether extract; NDF, neutral detergent fiber; ADF, acid detergent fiber; ADL, acid detergent lignin; AC, *Acremonium cellulase* (Meiji Seika Pharma Co. Ltd., Tokyo, Japan); TC, *Trichoderma cellulase* (Meiji Seika Pharma Co. Ltd., Japan); SEM, standard error of the mean.

a–l Means within columns with different superscripts differ at p<0.05.

Values are means of three silage samples.

**Table 6:** *In vitro* dry matter digestibility (IVDMD, % of DM) and *in vitro* organic matter digestibility (IVOMD, % of DM) at 6 and 48h of incubation of Guinea grass and Napier grass silages at 30 d after fermentation

Treatments	IVDMD		IVOMD	
	
	6 h	48 h	6 h	48 h
Fresh Guinea				
AC 0.0025%	30.02abcdef	55.62fgh^ij^	36.98bcd	64.85^ef^
AC 0.005%	30.46abcdef	54.85^gh^^ij^	36.01^cd^	64.38^ef^
AC 0.01%	32.21abcd	60.76cdefgh	42.65abc	67.17cdef
TC 0.0025%	26.69cdef	54.05^ij^	36.29^cd^	64.09^ef^
TC 0.005%	25.16^ef^	54.43^h^^ij^	35.81^cd^	64.54^ef^
TC 0.01%	26.36def	52.19^j^	36.06^cd^	61.68^f^
Wilted Guinea				
AC 0.0025%	27.99bcdef	59.48defgh^i^	38.17bcd	68.73bcde
AC 0.005%	31.14abcdef	57.38efgh^ij^	40.96abcd	66.62def
AC 0.01%	34.59^ab^	59.44defgh^i^	44.23^ab^	71.89abcd
TC 0.0025%	24.83^f^	57.12efgh^ij^	34.91^d^	66.21def
TC 0.005%	32.68abcd	58.51defgh^i^	39.29bcd	68.12bcde
TC 0.01%	29.52abcdef	59.23defgh^i^	38.83bcd	68.23bcde
Fresh Napier				
AC 0.0025%	29.12abcdef	57.35efgh^ij^	38.57bcd	67.31cdef
AC 0.005%	33.22abcd	66.26abc	42.98abc	73.32abc
AC 0.01%	35.60^a^	69.68^a^	41.33abcd	75.77^a^
TC 0.0025%	33.87^ab^	68.12^ab^	41.14abcd	73.74^ab^
TC 0.005%	30.19abcdef	61.45cdef	38.92bcd	72.08abcd
TC 0.01%	31.96abcde	62.42bcde	40.61abcd	73.35abc
Wilted Napier				
AC 0.0025%	32.47abcd	62.34bcde	42.58abc	71.56abcd
AC 0.005%	33.53abc	64.55abcd	41.62abcd	73.84^ab^
AC 0.01%	35.02^ab^	66.69abc	47.15^a^	76.07^a^
TC 0.0025%	31.77abcdef	63.98abcd	40.71abcd	73.38abc
TC 0.005%	34.15^ab^	61.13cdefg	42.76abc	71.10abcd
TC 0.01%	32.94abcd	64.84abcd	41.65abcd	74.19^ab^
SEM	2.392	2.184	2.499	2.165
Grasses means				
Guinea grass	29.31^b^	38.35^b^	56.92^b^	66.38^b^
Napier grass	32.82^a^	41.67^a^	64.07^a^	72.97^a^
Moisture adjust				
Fresh	30.41	59.76	38.95^b^	68.52^b^
Wilted	31.72	61.22	41.07^a^	70.83^a^
Enzymes				
AC	32.11^a^	61.20	41.10^a^	70.12
TC	30.01^b^	59.79	38.92^b^	69.23
Application levels				
0.0025%	29.60^b^	59.76^b^	38.67^b^	68.73^b^
0.005%	31.32^ab^	59.82^b^	39.79^ab^	69.25^ab^
0.01%	32.27^a^	61.90^a^	41.56^a^	71.04^a^
Significance of main effect and interaction				
Grasses means (A)	<0.001	<0.001	<0.001	<0.001
Moisture adjust (B)	0.127	0.065	0.020	0.004
Enzymes (C)	0.016	0.074	0.017	0.246
Application levels (D)	0.040	0.044	0.034	0.044
A×B×C×D	0.445	<0.001	0.787	0.264

AC, *Acremonium* cellulase (Meiji Seika Pharma Co. Ltd., Tokyo, Japan); TC, *Trichoderma* cellulase (Meiji Seika Pharma Co. Ltd., Japan); SEM, standard error of the mean.

a–h Means within columns with different superscripts differ at p<0.05.

Values are means of three silage samples.
